# Eco-friendly simultaneous multi-spectrophotometric estimation of the newly approved drug combination of celecoxib and tramadol hydrochloride tablets in its dosage form

**DOI:** 10.1038/s41598-023-38702-9

**Published:** 2023-07-20

**Authors:** Hesham Sameh Ramadan, Randa A. Abdel Salam, Ghada M. Hadad, Fathalla Belal, Mohamed M. Salim

**Affiliations:** 1 Department of Pharmaceutical Analytical Chemistry, Faculty of Pharmacy, Suez Canal University, Ismailia, Egypt; 2 Department of Pharmaceutical Chemistry, Faculty of Pharmacy, Horus University- Egypt, New Damietta, Egypt; 3 Department of Pharmaceutical Analytical Chemistry, Faculty of Pharmacy, Mansoura University, Mansoura, 35516 Egypt

**Keywords:** Analytical chemistry, Green chemistry

## Abstract

Food and Drug Administration (FDA) recently approved co-formulated celecoxib and tramadol for the treatment of acute pain in adults. Three spectrophotometric methods were efficiently applied to estimate the co-formulated Celecoxib and Tramadol in their tablets; second derivative 2D-spectrophotometry technique (method I), induced dual-wavelength technique (method II) and dual-wavelength resolution technique (method III). The proposed methods were successfully validated following the International Council for Harmonisation (ICH) guidelines and statistically assessed based on the correlation coefficients, relative standard deviations as well as detection and quantitation limits. The obtained results revealed non-significant differences compared to the reported results as revealed by the variance ratio F test and Student t test. Moreover, the applied techniques were further assessed concerning their greenness based on the analytical eco-scale method revealing an excellent green scale with a final score of 95. The proposed spectrophotometric techniques could be applied for the routine analysis and quality control of the studied drugs in their dosage form.

## Introduction

**Figure 1:**
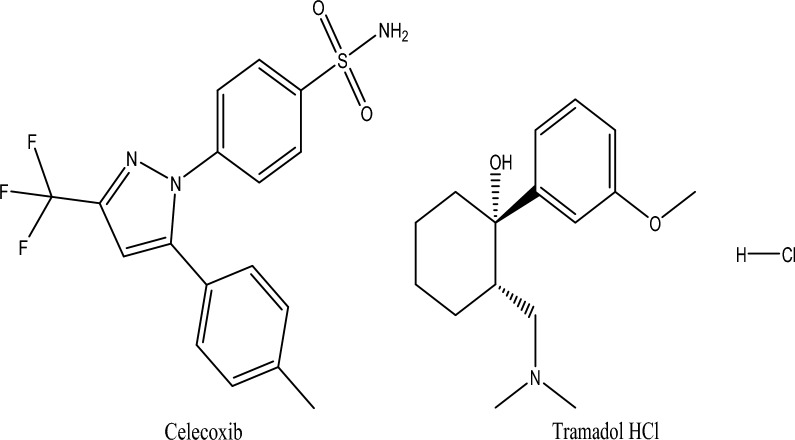
Chemical Structures of the studied drugs.

Celecoxib (4-[5-(4-methylphenyl)-3-(trifluoromethyl)-1H-pyrazol-1-yl] benzene sulfonamide) (Fig. 1) is a COX-2 selective NSAID, approved for the treatment of patients suffering from rheumatism and osteoarthritis^1^. It is considered an alternative drug to curtail the upper gastrointestinal toxicity of non-selective NSAIDs and can be prescribed in rheumatoid arthritis or for osteoarthritic patients as a first-line NSAID^2^. Other studies suggest its potential use against numerous cancers, whether in combination with other drugs^3^ or via modification of its chemical structure and creation of different analogs^4^. Several reported methods of analysis for Celecoxib are found in the literature whether alone or with other drugs, including spectrophotometric methods^5–7^, HPLC^8–11^, TLC^12–14^, and electrochemical methods^15–17^.

Tramadol (trans‐2‐ [(dimethylamino) methyl] ‐1‐ (3‐methoxyphenyl) cyclohexanol) (Fig. 1) is a centrally acting analgesic drug utilized for the treatment of moderate to severe pain. Caution must be exercised upon the usage of tramadol because of its potential for substantial abuse and adverse side effects^18^. Recently, Tramadol was suggested as being beneficial in the treatment of COVID-19^19^. Numerous methods were reported for the analysis of Tramadol either alone or with different drugs, viz*.* HPLC^20–25^, HPTLC^26^, Gas Chromatography^27,28^, spectrophotometric methods^29–31^, electrochemical methods^32–35^, and capillary electrophoresis^36,37^.

Pharmaceutical cocrystals are a novel class of engineered solid forms and are one of the approaches for ameliorating the physicochemical and biopharmaceutical properties of drugs with no change in their chemical structures^38,39^. The mixture of interest was first identified as a novel active pharmaceutical ingredient (API-API) co-crystal formed by an intrinsic 1:1 molecular ratio of racemic-tramadol.HCl and celecoxib^40^ were found to produce antinociceptive effects in a postoperative pain model in rats^41^. Some clinical trials were applied and found that the mixture may provide an appropriate addition to pain therapy because of its unique structure and improved pharmacokinetics in addition to its higher risk–benefit ratio^42–44^. Furthermore, the co-formulation approach offers several advantages, such as low medication errors and augmented convenience for patients, albeit the disadvantages of increased complexity in drug development, characterization, and quality control^45^. An oral tablet dosage form each containing 56 mg of celecoxib as well as 44 mg of tramadol was later approved in October 2021^46^. The preparation is indicated for the management of acute pain in adults in cases in which other alternative treatments have proven inadequate or not tolerated by patients^47^.

To the best of our knowledge, only one spectrophotometric method for the assessment of the aforementioned mixture was reported so far^48^. In addition, the complete overlap of spectra of both drugs poses a challenge for the simultaneous estimation of both drugs. This paper presents three spectrophotometric methods; 2nd derivative, induced dual-wavelength, and dual-wavelength resolution techniques for the simultaneous assay of celecoxib and tramadol in their commercial product. The proposed method is successfully applied for the simultaneous estimation of the studied drugs in dosage form with better LOD and LOQ for tramadol. In addition, using the 2nd derivative technique requires minimal mathematical manipulation than the others. Moreover, the IDW technique is considered relatively new.

An expanded attentiveness to the implementation of green chemistry principles in analytical techniques has grown vastly^49^. For assessing the greenness of analytical techniques, several methods are employed viz. the Analytical Eco-Scale Metric that considers various features of the analytical technique to calculate the procedure's green index^50^. The assessment of our applied techniques using the Analytical Eco-Scale Metric was therefore employed for the determination of their greenness indices.

## Theoretical background

### Method I: induced dual wavelength method (IDW)

This method is intended for application to a binary mixture (X and Y) having completely overlapped zero-order absorption spectra at two wavelengths λ_1_ and λ_2_ respectively, in which the absorbance of the interfering substances at the selected wavelengths are not equal (so that absorbance difference at the said wavelengths is not equal to zero), rendering the conventional dual-wavelength method inapplicable^51^. The technique is relatively new and was utilized a few times to resolve some challenging components in mixtures^5,51–57^. Briefly, the equations describing this situation are as follows:

1 $${\text{A}}_{{1}} = {\text{A}}_{{{\text{X1}}}} + {\text{ A}}_{{{\text{Y1}}}} \quad \quad \quad \quad {\text{at}}\;{\uplambda }_{{1}}$$

2 $${\text{A}}_{{2}} = {\text{A}}_{{{\text{X2}}}} + {\text{A}}_{{{\text{Y2}}}} \quad \quad \quad \quad {\text{at}}\;{\uplambda }_{{2}}$$

where

A_1_ is the mixture absorbance at λ_1_ which is chosen as the λ_max_ of X.

A_2_ is the mixture absorbance at λ_2_ which is any other wavelength.

An equality factor is calculated for component Y to eliminate its effect at the two wavelengths as follows:

$${\text{F}}_{{\text{Y}}} = {\text{A}}_{{{\text{Y1}}}} {\text{/A}}_{{{\text{Y2}}}} \quad \quad \quad \quad \therefore {\text{A}}_{{{\text{Y1}}}} = {\text{F}}_{{\text{Y}}} {\text{A}}_{{{\text{Y2}}}}$$

Substitution in Eq. (1) gives:

3 $${\text{A}}_{{1}} = {\text{ A}}_{{{\text{X1}}}} + {\text{ F}}_{{\text{Y}}} {\text{A}}_{{{\text{Y2}}}}$$

Then multiplying Eq. (2) by the equality factor F_Y_ gives:

4 $${\text{F}}_{{\text{Y}}} {\text{A}}_{{2}} = {\text{ F}}_{{\text{Y}}} {\text{A}}_{{{\text{X2}}}} + {\text{ F}}_{{\text{Y}}} {\text{A}}_{{{\text{Y2}}}}$$

Subtraction of Eq. (4) from Eq. (3) gives:

5 $$\Delta {\text{A }}\left( {{\text{A}}_{{1}} - {\text{F}}_{{\text{Y}}} {\text{A}}_{{2}} } \right) = {\text{A}}_{{{\text{X1}}}} - {\text{F}}_{{\text{Y}}} {\text{A}}_{{{\text{X2}}}}$$

The inspection of Eq. (5) indicates the sole dependence of the absorbance difference of the mixture on the C_X_ with no interference from C_Y_. Thus, the concentration of component X can be computed from the following regression equation:

6 $$\left( {\Delta {\text{A }} = {\text{ A}}_{{1}} - {\text{ F}}_{{\text{Y}}} {\text{A}}_{{2}} } \right) \, = {\text{ slope}}.{\text{C}}_{{\text{X}}} \pm {\text{ intercept}}$$

Via plotting the absorbance difference values of the zero-order spectra of pure X at the selected wavelengths (∆A = A_1_ − F_Y_A_2_) versus the corresponding concentrations of X, the corresponding regression equation can be obtained.

### Method II: dual wavelength resolution technique (DWRT)

This method is applied when the IDW method is inapplicable to determine the concentration of the other component Y^51^. After the calculation of the concentration of the 1st component X using the IDW technique, the zero-order spectrum of component X is acquired by multiplication of the calculated concentration by the normalized absorptivity curve of component Y. The normalized absorptivity curve of component X is obtained by the division of the whole spectrum of X by its corresponding concentration giving a spectrum indicating the absorptivity of the analyte of interest (a_X_) against all the measured wavelengths. The next step is obtaining the spectrum of component Y by subtracting the calculated spectrum of X from the spectrum of the corresponding mixture. Due to the problem of obtaining indefinite peaks in the zero-order spectrum of Y, the first derivative spectrum was obtained as a result and the corresponding regression equation was computed.

### Method III: 2nd derivative technique

The 2nd derivative spectrophotometry was successfully applied for the simultaneous estimation of both drugs without a prior separation step. This method has the advantage of the evaluation of complex mixtures without chemical treatment. However, difficulties may sometimes arise as the signal-to-noise ratio can be reduced, in addition to the complex profiles obtained^58^.

## Experimental

### Apparatus

A Shimadzu ultraviolet–visible (UV–Vis) 1601 recording Spectrophotometer (P/N 206-67001, Japan) over the range of 190–500 nm was utilized for all measurements. The path length of the used cuvettes was 1 cm. Spectral mathematical manipulation was performed using UVProbe 2.42 software.

### Pure standards

Tramadol HCl was obtained from Fluka BioChemika, Buchs, Switzerland, with labeled purity of 99.0%.

Celecoxib with labeled purity of 99.44% was obtained from Amoun Pharmaceutical Co. (Cairo, Egypt).

Excipients used in the prepared tablets include talc, starch, magnesium stearate, avicel PH 112 FMC, and gelatin. All excipients were obtained from Sigma Co. for Pharmaceutical Industries, Quesna, Egypt.

### Solvents and chemicals

Methanol, ethanol, and acetonitrile (HPLC grade) were purchased from Fisher Scientific (UK).

Sodium hydroxide and HCl were purchased from El Nasr Pharmaceutical Chemicals Company (ADWIC, Cairo, Egypt).

### Standard solutions

Stock solutions of each celecoxib and tramadol were prepared separately using methanol to reach the concentration of 100 μg/mL. Further working solutions were freshly prepared using distilled water in 10 mL volumetric flasks as needed.

### Procedure

#### Spectral characteristics

Working solutions of celecoxib and tramadol were prepared separately by transferring appropriate volumes from corresponding stock solutions in 10 mL volumetric flasks. The volumes were completed to the mark using distilled water as a solvent. The blank used was distilled water and methanol in the ratio 1:1. The zero-order spectra of each compound were measured within the range 190–500 nm and stored in the computer.

#### Application of second derivative spectrophotometry

Series of different concentrations (2, 2.5, 3, 5, 7, 9, 12, 17, and 20 μg/mL) of celecoxib were prepared by transferring different volumes from stock solution into 10 mL volumetric flasks and performing dilution with distilled water to the mark. Similarly, a series of different concentrations (5, 7, 12, 15, 17, 25, 30, 35, 40, 45, and 50 μg/mL) of tramadol HCl were prepared using distilled water for dilution. The absorption spectra of each concentration were measured against a blank consisting of distilled water and methanol in the ratio of 1:1 and then were stored in the computer. Then, each spectrum was smoothed (∆λ = 4 nm) and the second derivative of each spectrum was obtained (∆λ = 4 nm, scaling factor = 100). The chosen wavelengths were 269.45 nm and 295.58 nm for tramadol and celecoxib, respectively. The calibration curve was constructed by plotting the amplitudes at the chosen wavelengths versus the corresponding concentrations and the regression equation was subsequently obtained.

#### Application of induced dual wavelength (IDW) technique

Series of concentrations (6, 8, 9, 10, 11.5, 13, 14, 15, 17, 18, and 20 μg/mL) were prepared as previously described in accordance with the ratio of celecoxib in dosage forms (5.5 tramadol: 7 celecoxib). The absorbance at wavelengths 250 nm and 271 nm was recorded. The equality factor required to diminish the effect of Tramadol in the mixture was calculated by measuring the absorbance of the corresponding tramadol HCl concentrations at the same wavelengths and dividing the first absorbance reading (250 nm) by the second one (271 nm). Afterward, the absorbance of celecoxib at 271 nm was multiplied by this equality factor, and the result was subtracted from the absorbance at 250 nm to obtain **∆A.** The calibration curve was constructed by plotting **∆A** against the corresponding concentration and the regression equation was computed.

#### Application of dual wavelength resolution technique (DWR)

Firstly, the normalized absorptivity curve for celecoxib was calculated by dividing the absorption spectrum of the drug by its corresponding concentration. A number of 11 normalized absorptivity curves was obtained and the average absorptivity curve was calculated. Secondly, the calculated absorption spectrum of celecoxib was obtained (after calculation of the concentration using the previous method) by multiplication of the calculated concentration of celecoxib by its average normalized absorptivity curve. After that, the corresponding absorption spectrum of tramadol was obtained by subtracting the calculated spectrum of celecoxib from the whole spectrum of the mixture. Finally, the obtained spectrum was smoothed (∆λ = 4 nm) and the first derivative was obtained (∆λ = 4 nm, scaling factor = 100). The absorbance of tramadol was then measured at 281.68 nm. The calibration curve was constructed by plotting the amplitudes at the chosen wavelengths against the corresponding concentrations and the regression equation was obtained.

#### Preparation of synthetic mixtures

A number of 11 mixtures was prepared with concentration ratios in the range of 5–15 μg/mL and 6–20 μg/mL for Tramadol and Celecoxib, respectively for the construction of calibration curves for both the Induced dual wavelength (IDW) and Dual wavelength resolution (DWRT) techniques. For the 2nd derivative technique, Celecoxib concentrations of 2, 2.5, 3, 5, 7, 9, 12, 17, and 20 μg/mL and Tramadol HCl concentrations of 5, 7, 12, 15, 17, 25, 30, 35, 40, 45and 50 μg/mL were used for the construction of calibration curves.

#### Laboratory-prepared tablets

Since the dosage form is not yet available in the Egyptian market, the laboratory-prepared tablet was used. Formula per tablet was prepared by weighing 56 mg of celecoxib, 44 mg of tramadol HCl, talc, starch, magnesium stearate, gelatin, and Avicel pH 112 FMC. The ingredients were triturated in a porcelain mortar and then transferred to a 100 mL volumetric flask. Around 60 mL of methanol was added. After sonication for 30 min, the volume was completed to 100 mL with the same solvent. The solution was subsequently double-filtered using Whatmann no. 1 filter paper. Aliquot volumes were transferred to 10 mL measuring flasks and completed to the mark with distilled water to reach the following concentration ratios (7:9, 9:11.5, and 10:13 tramadol to celecoxib, respectively).

#### Evaluation of the method greenness

The method greenness was assessed based on the analytical Eco-scale approach which utilizes attributing penalty points to parameters that don’t abide by the ideal green analysis. The analytical eco-scale was obtained using the following equation^49^:

$${\text{Analytical}}\;{\text{Eco-Scale}} = {1}00 - {\text{total}}\;{\text{penalty}}\;{\text{points}}{.}$$

## Results and discussion

**Figure 2:**
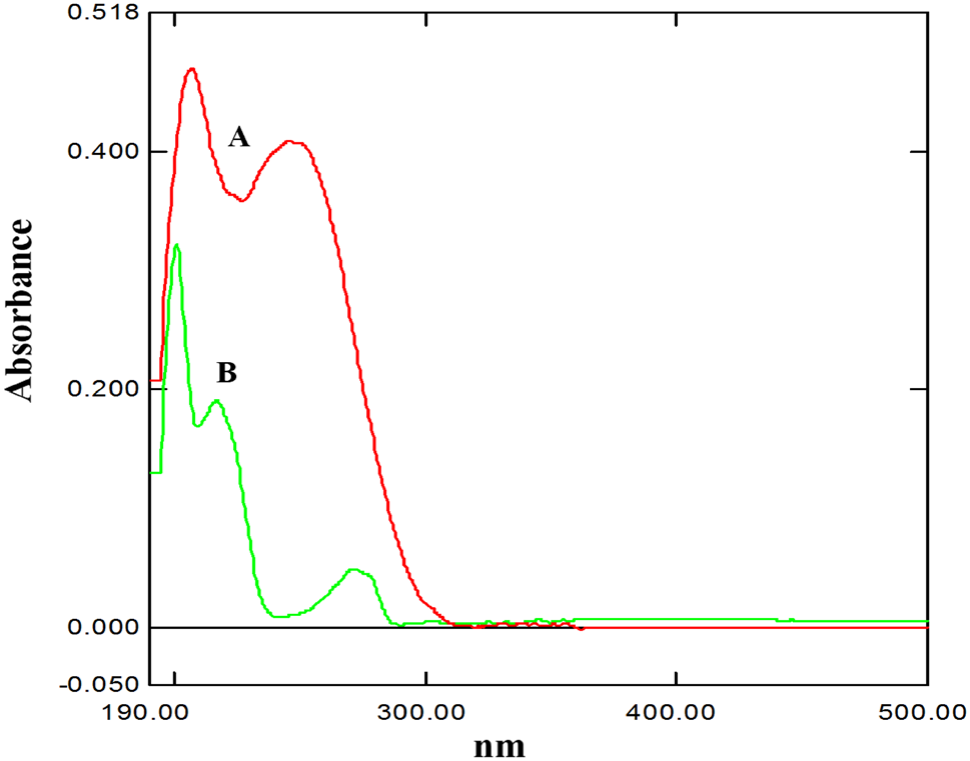
Zero order spectra of celecoxib (**A**) and tramadol HCl (**B**) (10.0 μg/mL) in distilled water.

The completely overlapped spectra of the two drugs represented a challenge for their estimation (Fig. 2), hampering the use of other conventional methods such as the ratio spectra, ratio derivative, etc.

**Figure 3:**
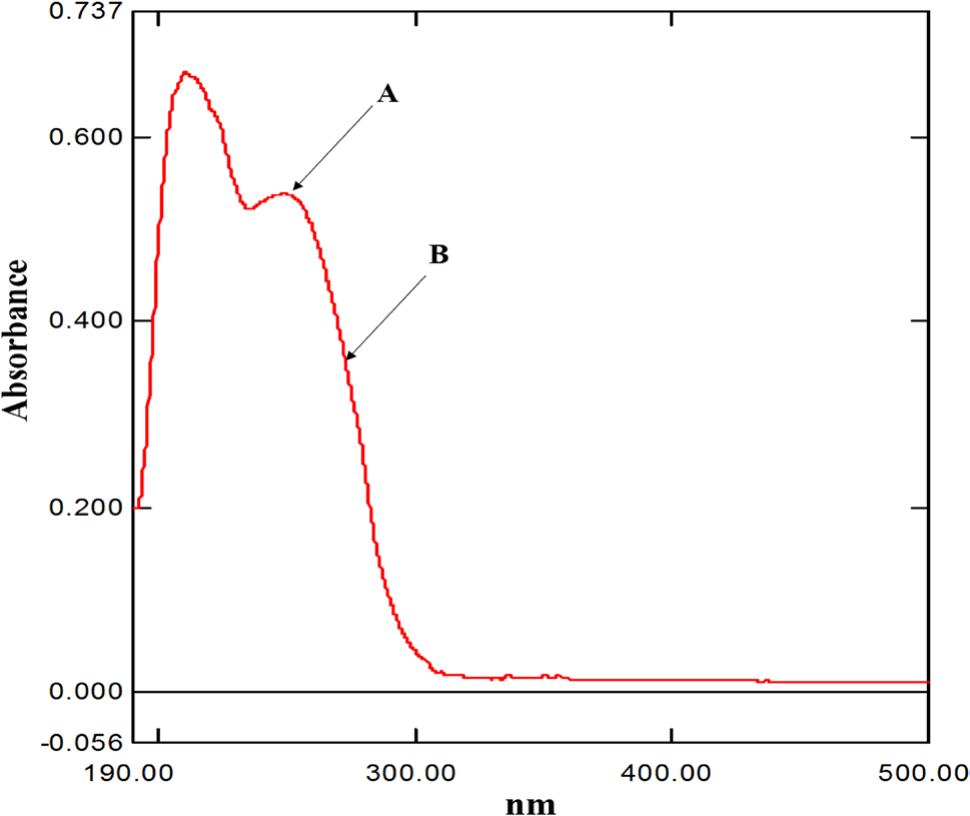
Chosen wavelengths (**A**) (250 nm) and (**B**) (271 nm) for application of Induced dual wavelength on a synthetic mixture containing celecoxib (11.5 μg/mL) and tramadol (9.0 μg/mL).

The IDW technique was successfully applied to resolve celecoxib in the mixture of interest (Fig. 3).

**Figure 4:**
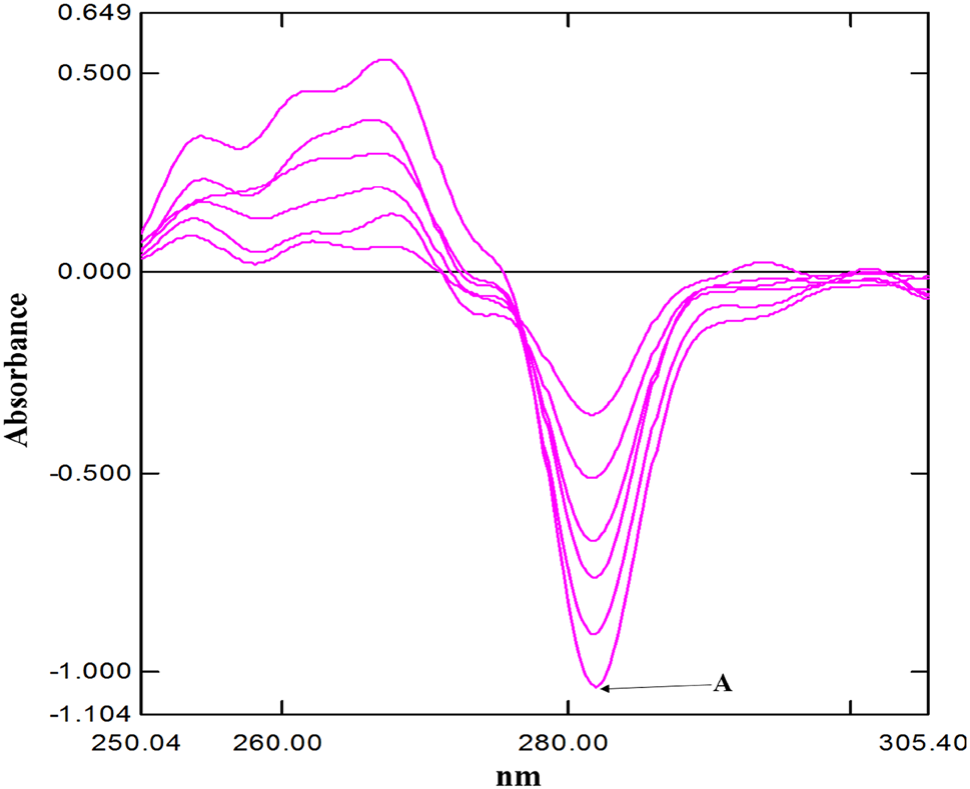
Obtained 1st derivative spectra of Tramadol HCl using dual wavelength resolution measured at 281.68 nm (**A**) (concentrations 5.0, 7.0, 9.0, 11.0, 13.0 and 15.0 μg/mL).

The DWRT was used for the estimation of tramadol as the IDW technique was inapplicable (Fig. 4).

**Figure 5:**
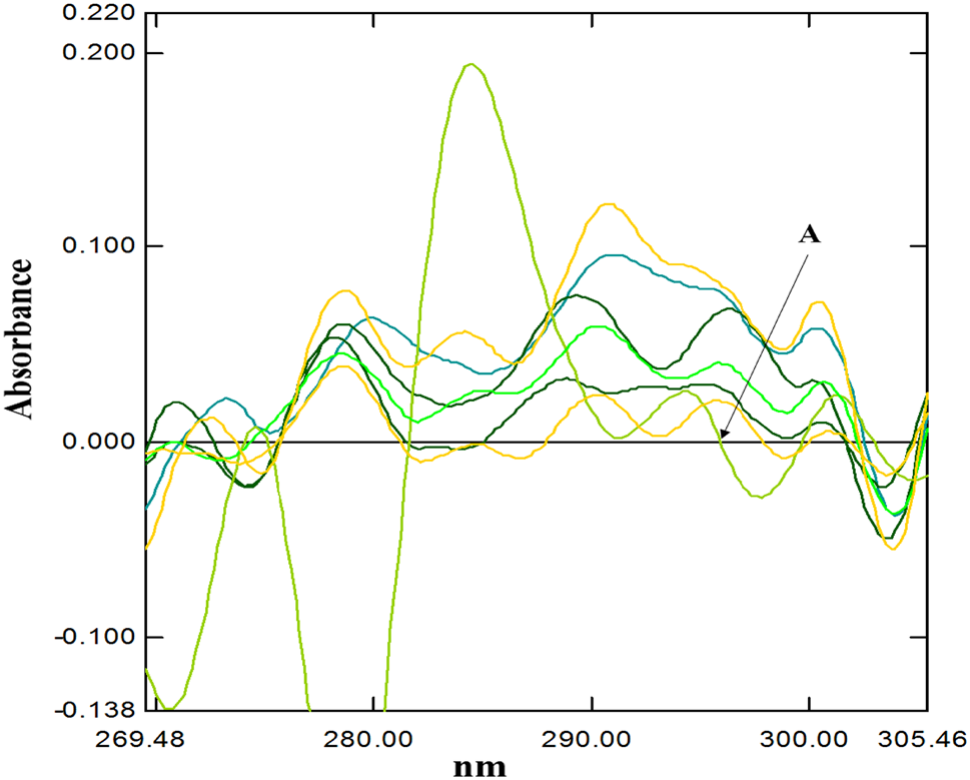
2nd derivative spectra indicating Zero crossing point of tramadol HCl (concentration 15.0 μg/mL) (**A**) in the presence of Celecoxib (concentration 2.0, 5.0, 9.0, 12.0, 17.0, 20.0 μg/mL) at 295.58 nm.

**Figure 6:**
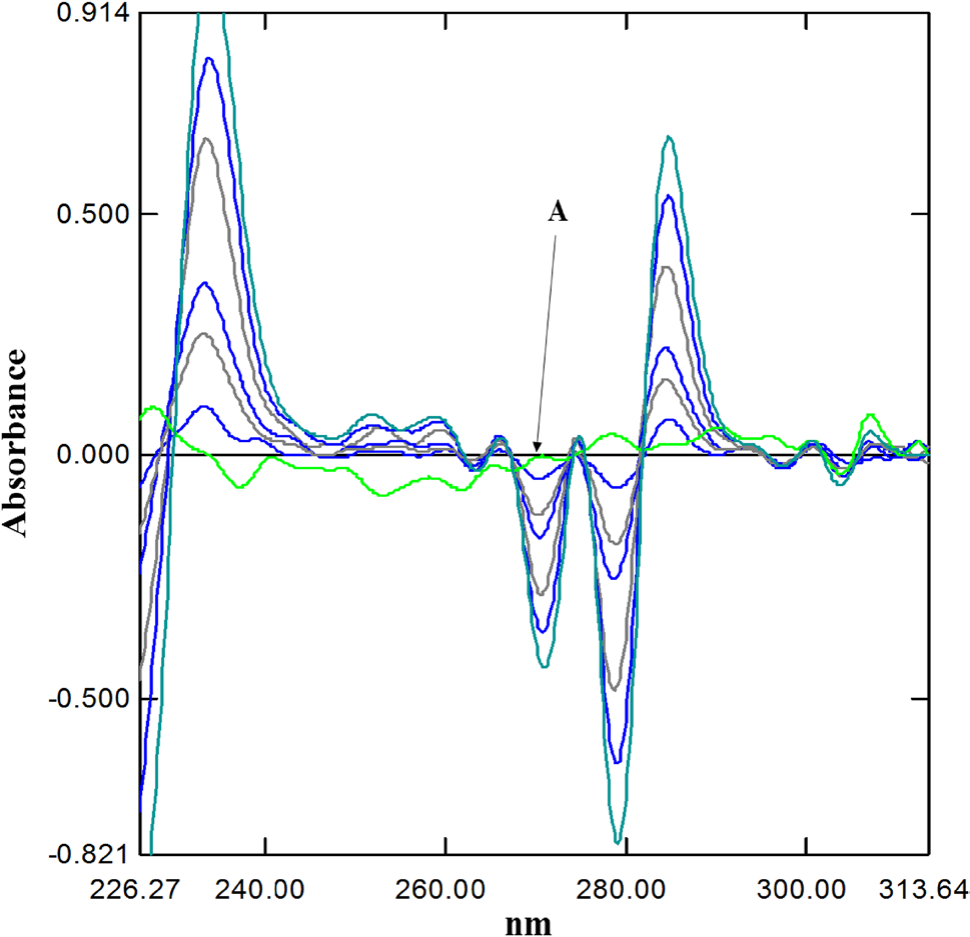
2nd derivative spectra indicating Zero crossing point of Celecoxib (concentration 9 μg/mL) (**A**) in the presence of Tramadol HCl (concentrations 5.0, 12.0, 17.0, 30.0, 40.0, 50.0 μg/mL) at 269.45 nm.

The 2nd derivative technique was successfully applied for estimation of both drugs in their binary mixtures (Figs. 5, 6).

### Optimization of experimental conditions

#### Effect of various solvents

Different solvents such as methanol, ethanol, and acetonitrile in addition to 0.1N HCl and 0.1N NaOH were used. No significant difference was found from spectra obtained in water, so distilled water was the solvent of choice due to its availability, cheap cost, and environmental safety.

### Analytical performance and method validation

The methods were validated in accordance with the ICH guidelines^59^. The data indicated that the methods were of acceptable accuracy, precision, and specificity over the specified linearity range.

#### Linearity and range

The linear range as well as the correlation coefficient are calculated and the obtained results are summarized in Table 1. The linearity was evaluated by the least squares method indicating acceptable accuracy in accordance with ICH guidelines^59^.

**Table 1:** Performance data of the proposed methods.

Parameters	Celecoxib		Tramadol HCl	
	2nd Derivative method	IDW method	2nd derivative method	DWR method
Linearity (μg/mL)	2.0–20.0	6–20	5.0–50.0	5–15
LOD (μg/mL)	0.313	0.436	0.388	0.3128
LOQ (μg/mL)	0.950	1.32	1.176	0.9478
Correlation coefficient (r)	0.9998	0.9996	− 0.9999	− 0.9996
Slope	0.0039	0.0375	− 0.009	− 0.0635
Intercept	0.0124	0.0275	− 0.0038	− 0.0551
S_y/x_ (standard deviation of residuals)	6.40 × 10^−4^	4.90 × 10^−3^	1.77 × 10^−3^	6.02 × 10^−3^
S_a_ (standard deviation of the intercept of the regression line)	3.70 × 10^−4^	4.95 × 10^−3^	1.06 × 10^−3^	6.02 × 10^−3^
S_b_ (standard deviation of the slope of the regression line)	3.00 × 10^−5^	3.70 × 10^−4^	4.00 × 10^−5^	5.70 × 10^−4^
% Error	0.32	0.33	0.32	0.23
% RSD	0.96	1.09	1.04	0.77
Mean Found (%) ± SD	100.29 ± 0.962	99.90 ± 1.086	99.90 ± 1.035	99.98 ± 0.770

#### Limits of detection (LOD) and limits of quantification (LOQ)

The Limit of Detection (LOD) and limit of quantification (LOQ) for each method were mathematically computed following ICH guidelines^59^ and the data obtained are abridged in Table 1. The LOD was calculated from the following equation:

$$LOD=\frac{3.3\sigma }{s}$$

While the LOQ was calculated from the following equation:

$$LOQ=\frac{10\sigma }{s}$$

where σ is the standard deviation of the response and s is the slope of the calibration curve estimated from the regression line. The ICH guidelines mandate the signal-to-noise ratio to be equal to 3:1 and 10:1 in the case of LOD and LOQ, respectively^59^.

#### Precision and accuracy

For the estimation of accuracy, the results of the proposed methods were compared to those obtained from previously reported methods following the guidelines of ICH using three replicate results for three different concentrations within the linear range^59^. For tramadol HCl, it was determined in methanol through its zero order spectra at λ_max_ = 275 nm^60^, whereas for celecoxib, it was determined in 0.1N NaOH as solvent through its 1st order spectra at λ_max_ = 269 nm^61^. These methods were also applied to the dosage forms and no significant difference was found as per the values of t test and F values^62^ (Table 2).

**Table 2:** Statistical comparison between the proposed methods and reference methods ^60,61^ of pure drugs.

Parameters	Celecoxib			Tramadol		
	2nd Derivative	IDW technique	Reference method^61^	2nd Derivative	DWR method	Reference method^60^
Prepared tablets						
Mean % Recoveries ± SD	101.86 ± 0.054	101.91 ± 0.069	101.85 ± 0.039	101.61 ± 0.497	101.25 ± 0.796	102.06 ± 1.062
% Error	0.031	0.039	0.017	0.282	0.454	0.454
% RSD	0.053	0.068	0.038	0.489	0.786	0.786
t test	0.354 (2.447)*	1.795 (2.447)*	–	0.915 (2.776)*	1.334 (2.776)*	–
F value	1.958 (6.944)*	3.163 (6.944)*	–	1.972 (19)*	1.2997 (19)*	–
Pure drugs						
Linearity range (μg/mL)	2–20	6–20	1–20	5–50	5–15	10–100
Mean % Recoveries ± SD	100.29 ± 0.962	99.90 ± 1.086	99.35 ± 1.444	99.90 ± 1.035	99.98 ± 0.77	99.61 ± 0.818
% Error	0.321	0.327	0.646	0.312	0.232	0.366
% RSD	0.959	1.087	1.453	1.036	0.77	0.821
t test	1.465 (2.179)*	0.837 (2.145)*	–	0.544 (2.145)*	0.863 (2.145)*	–
F value	3.304 (4.347)*	1.7695 (3.478)*	–	1.603 (5.964)*	1.128 (3.478)*	–

*The values between parentheses indicate the tabulated t and F values at *P* = 0.05^62^.

Intra and Inter day precision were studied by applying the proposed methods for the determination of three concentrations within the studied ranges on three successive times or three successive days in accordance with the ICH guidelines^59^. (Table 3).

**Table 3:** Precision data of the proposed methods.

Parameters	Celecoxib				Tramadol HCl			
	2nd Derivative method		IDW method		2nd Derivative method		DWR method	
	Amount taken (μg/mL)	% Recoveries *	Amount taken (μg/mL)	% Recoveries *	Amount taken (μg/mL)	% Recoveries *	Amount taken (μg/mL)	% Recoveries *
Intraday precision	5	101.15	7	98.83	7	101.79	7	100.24
		101.92		102.88		101.07		101.65
		101.54		103.27		100.89		98.11
	Mean % ± SD	101.54 ± 0.385	Mean % ± SD	101.66 ± 2.458	Mean % ± SD	101.25 ± 0.472	Mean % ± SD	100 ± 1.785
	% Error	0.219	% Error	1.396	% Error	0.269	% Error	1.031
	% RSD	0.379	% RSD	2.418	% RSD	0.466	% RSD	1.785
	7	100.71	9	98.02	9	101.35	9	100.69
		101.19		99.26		101.89		100.17
		99.76		99.72		101.22		99.13
	Mean % ± SD	100.56 ± 0.727	Mean % ± SD	99.00 ± 0.881	Mean % ± SD	101.49 ± 0.358	Mean % ± SD	100 ± 0.794
	% Error	0.417	% Error	0.514	% Error	0.204	% Error	0.458
	% RSD	0.723	% RSD	0.89	% RSD	0.353	% RSD	0.794
	9	101.6	13	101.41	12	101.79	12	100.75
		99.6		102.94		99.11		100.88
		101.2		102.39		100.89		100.50
	Mean % ± SD	100.8 ± 1.058	Mean % ± SD	102.24 ± 0.774	Mean % ± SD	100.60 ± 1.364	Mean % ± SD	100.71 ± 0.192
	% Error	0.606	% Error	0.437	% Error	0.783	% Error	0.11
Interday precision	5	99.63	7	100.24	7	100.69	7	99.76
		101.11		98.42		101.39		102.13
		98.89		100.79		99.31		100.24
	Mean % ± SD	99.88 ± 1.132	Mean % ± SD	99.82 ± 1.244	Mean % ± SD	100.46 ± 1.061	Mean % ± SD	100.71 ± 1.251
	% Error	0.654	% Error	0.719	% Error	0.61	% Error	0.717
	% RSD	1.133	% RSD	1.246	% RSD	1.056	% RSD	1.242
	7	99.29	9	101.37	9	101.03	9	101.21
		101.19		97.77		98.97		100.69
		98.81		102.27		101.03		99.48
	Mean % ± SD	99.76 ± 1.26	Mean % ± SD	100.47 ± 2.38	Mean % ± SD	99.66 ± 1.19	Mean % ± SD	100.46 ± 0.889
	% Error	0.729	% Error	1.368	% Error	0.685	% Error	0.511
	% RSD	1.263	% RSD	2.369	% RSD	1.186	% RSD	0.885
	9	101	13	101.78	12	99.21	12	97.86
		99		98.35		100.79		98.74
		101.2		101.91		99.61		101.13
	Mean % ± SD	100.4 ± 1.217	Mean % ± SD	100.68 ± 2.02	Mean % ± SD	99.87 ± 0.82	Mean % ± SD	99.25 ± 1.692
	% Error	0.7	% Error	1.158	% Error	0.474	% Error	0.984
	% RSD	1.212	% RSD	2.006	% RSD	0.821	% RSD	1.705

*Average of three determinations for each concentration.

#### Robustness

Distilled water was used for simplicity throughout the determination. No significant change was found when using different pH such as 0.1N NaOH or 0.1N HCl, so no buffer was used during the procedure. The robustness of the experimental procedure is thus confirmed as it was proven reliable regarding deliberate variations in parameters in agreement with ICH guidelines^59^.

#### Selectivity

Using the 2nd derivative technique, celecoxib was determined at λ_max_ = 295.58 nm with no interference from tramadol HCl. Likewise, tramadol was determined at λ_max_ = 269.45 nm with no interference from celecoxib, indicating acceptable Selectivity. Furthermore, Using the IDW technique, the selectivity was enhanced and the interference from tramadol HCl was completely diminished by the equality factor, leading to the successful determination of celecoxib. Additionally, tramadol was successfully determined using DWRT with no interference from celecoxib. The excipients added to the laboratory-prepared tablets showed no interference with the applied techniques. Based on these findings, these methods provide good selectivity for the simultaneous determination of both drugs in a synthetic mixture and prepared tablet in the presence of excipients according to the requirements of the ICH^59^.

#### Application on laboratory prepared tablets

Table 4 shows the data of the proposed methods applied to the laboratory-prepared tablet. The results indicate that the proposed methods have acceptable accuracy and precision with respect to both drugs under investigation. In addition, no significant interference from the excipients in the prepared tablets was found, indicating acceptable selectivity for both drugs in the laboratory-prepared tablets.

**Table 4:** Application of the proposed methods to prepared tablets.

Parameters	Celecoxib				Tramadol HCl			
	2nd Derivative method		IDW method		2nd Derivative method		DWR method	
	Amount taken (μg/mL)	% Recoveries*	Amount taken (μg/mL)	% Recoveries*	Amount taken (μg/mL)	% Recoveries*	Amount taken (μg/mL)	% Recoveries*
Prepared tablets	9	101.88	9	101.98	7	101.56	7	102.03
	11.5	101.80	11.5	101.93	9	101.36	9	101.27
	13	101.90	13	101.84	10	102.13	10	100.44
Mean % ± SD	101.86 ± 0.054		101.92 ± 0.069		101.68 ± 0.497		101.25 ± 0.796	
% Error	0.031		0.039		0.282		0.454	
% RSD	0.053		0.068		0.489		0.786	

* Average of three determinations for each concentration.

#### Evaluation of the method greenness according to the analytical eco-scale approach

The concept of green chemistry aims at reducing both using and generating toxic hazardous materials. However, certain difficulties might arise upon evaluating the analytical methodologies such as the number of analytes to be determined, the techniques to be used as well as some important parameters needed to be considered such as LOD. In addition, the least green phase in the analytical process is sample preparation according to previously reported data^49^. Table 5 estimates the final score to be 95, indicating excellent green analysis techniques.

**Table 5:** Calculation of penalty points for the proposed methods with respect to the analytical Eco-Scale method.

The penalty points (PPs) calculation according to analytical eco-scale		Penalty points
Reagents		
Methanol : water (1:1)	Amount < 10 mL	2
Hazard (physical, environmental, health)	More severe hazard	
Instrument		
Energy	Technique: spectrophotometry ≤ 0.1 kWh per sample	0
Occupational hazard	Analytical process hermetization	0
Waste	1–10 mL (g)	3
Total penalty points		5
Analytical eco-scale total score *		95*

*Score ranking scale: > 75, Excellent green analysis; > 50, Acceptable green analysis; < 50, Inadequate green analysis^49^.

## Conclusion

The goal of this study was to implement three Eco-friendly spectrophotometric methods for the assessment of the synthetic mixtures in addition to laboratory-prepared tablets of the recently approved drug combination (celecoxib/tramadol HCl). The applied methods including; the second derivative 2D-spectrophotometry technique (method I), induced dual-wavelength technique (method II), and dual-wavelength resolution technique (method III) revealed acceptable accuracy, good linearity, reproducibility as well as precision and can be applied for the routine analysis and quality control of the co-formulated mixture. Further, the methods also have the additional advantages of speed and simplicity, in addition to environmental safety as they don’t require the use of hazardous solvents or sophisticated instruments. Statistical analysis has been accomplished illustrating non-significant differences compared to the previously reported data. Furthermore, the greenness index of the applied methods was measured via Eco-scale metric revealing an excellent green index of the methods employed.

The proposed study poses simple, rapid, and reliable methods for the analytical assessment of the newly approved mixture besides being Eco-friendly.

## Data Availability

All data generated or analyzed during this study are included in this published article.
